# Epigenetic silencing of microRNA-199b-5p is associated with acquired chemoresistance via activation of JAG1-Notch1 signaling in ovarian cancer

**DOI:** 10.18632/oncotarget.1458

**Published:** 2013-12-04

**Authors:** Michelle X. Liu, Michelle KY. Siu, Stephanie S. Liu, Judy WP. Yam, Hextan YS. Ngan, David W. Chan

**Affiliations:** ^1^ Department of Obstetrics and Gynaecology, LKS Faculty of Medicine, The University of Hong Kong, Hong Kong SAR, P.R.China; ^2^ Department of Pathology, LKS Faculty of Medicine, The University of Hong Kong, Hong Kong SAR, P.R.China

**Keywords:** MiR-199b-5p, JAG1-Notch1, acquired chemoresistance, epithelial ovarian cancer

## Abstract

Epithelial ovarian cancer is a highly lethal and aggressive gynecological malignancy. The high mortality rate is due in part to the fact that many advanced cancer patients become refractory to current chemotherapeutic agents, leading to tumor recurrence and death. However, the underlying mechanisms leading to chemoresistance remain obscure. Here, we report that the loss of miR-199b-5p due to progressive epigenetic silencing leads to the activation of the JAG1-mediated Notch1 signaling cascade, thereby leading to the development of acquired chemoresistance in ovarian cancer. Using miRCURY LNA™ microRNA array and Q-PCR analyses of two pairs of cisplatin-sensitive and –resistant ovarian cancer cell lines, we identified miR-199b-5p as significantly down-regulated in cisplatin-resistant ovarian cancer cells and confirmed that miR-199b-5p is clinically associated with advanced and poor survival ovarian cancers. Interestingly, the loss of miR-199b-5p could be restored by 5-Aza-dC-mediated demethylation, and methylated specific PCR (MS-PCR), bisulfite-sequencing and pyrosequencing revealed that the promoter region of miR-199b-5p was hypermethylated. Computational and mechanistic analyses identified JAG1 as a primary target of miR-199b-5p. Notably, the reduced expression of miR-199b-5p was found to be inversely correlated with the increased expression of JAG1 using an ovarian cancer tissue array. Enforced expression of miR-199b-5p sensitized ovarian cancer cells to cisplatin-induced cytotoxicity both *in vitro* and *in vivo*. Conversely, re-expression of miR-199b-5p and siRNA-mediated JAG1 knockdown or treatment with Notch specific inhibitor γ-secretase (GSI) attenuated JAG1-Notch1 signaling activity, thereby enhancing cisplatin-mediated cell cytotoxicity. Taken together, our study suggests that the epigenetic silencing of miR-199b-5p during tumor progression is significantly associated with acquired chemoresistance in ovarian cancer through the activation of JAG1-Notch1 signaling.

## INTRODUCTION

Epithelial ovarian cancer is one of the most lethal malignancies in females worldwide. The high mortality rate of this disease is due to its poor prognosis and the fact that the majority of patients are diagnosed at advanced stages. Therefore, chemotherapy combined with surgical cytoreduction is the standard initial management for ovarian cancer patients at advanced stages [1, 2]. Platinum-based chemotherapy is the standard first-line regimen for advanced ovarian cancer [3, 4]. The clinical response rate is initially high, but the subsequent relapse and repetitive challenges of chemotherapeutic agents leads to the development of acquired chemoresistance [5]. Such acquired chemoresistance is the major obstacle to the clinical management of ovarian cancer [6, 7]. However, the molecular mechanisms underlying acquired chemoresistance remain largely unknown, underlying the urgent need to identify the associated molecular mechanisms to explore alternative therapeutic strategies.

Acquired chemoresistance, also called as extrinsic chemoresistance, develops secondary to genetic and epigenetic alterations in cell proliferation, apoptosis, DNA repair, etc., after the administration of repetitive cycles of chemotherapy [5, 8, 9]. Genetic changes refer to the changes in the DNA sequence, including mutation, deletion, amplification, and translocation, whereas epigenetic alterations include DNA methylation, histone modifications and microRNA (miRNA) regulation. Emerging evidence suggests the importance of miRNAs in developing acquired chemoresistance in various human cancers [10-12]. The dysregulation of miRNAs alters a network of functional targets and signaling pathways, resulting in acquired chemoresistance in human cancers [10, 11]. For examples, up-regulation of miR-21 causes acquired trastuzumab resistance by repressing PTEN expression during long-term exposure to trastuzumab antibody in breast cancer [13], and the repetitive application of cisplatin (CDDP) to non-small cell lung cancer (NSCLC) A549 cells induces an increase in miR-630, which enhances cytoprotection against CDDP and carboplatin by inhibiting the p53 signaling pathway [14]. This evidence prompted us to hypothesize that some miRNAs may be involved in developing acquired chemoresistance during ovarian cancer progression.

In this study, we used the miRCURY LNA™ microRNA Array followed by a series of *in vitro* and *in vivo* functional and biochemical analyses to identify miR-199b-5p as being associated with cisplatin resistance in ovarian cancer cells. Importantly, our finding provides a novel molecular mechanism of epigenetic-mediated acquired chemoresistance in ovarian cancer by which miR-199b-5p is frequently silenced by DNA hypermethylation, thereby leading to the activation of JAG1-Notch1 signaling in ovarian cancer progression and acquired chemoresistance.

## RESULTS

### Identification of miR199b-5p as a putative miRNA candidate involved in the chemoresistance of ovarian cancer

**Fig 1:**
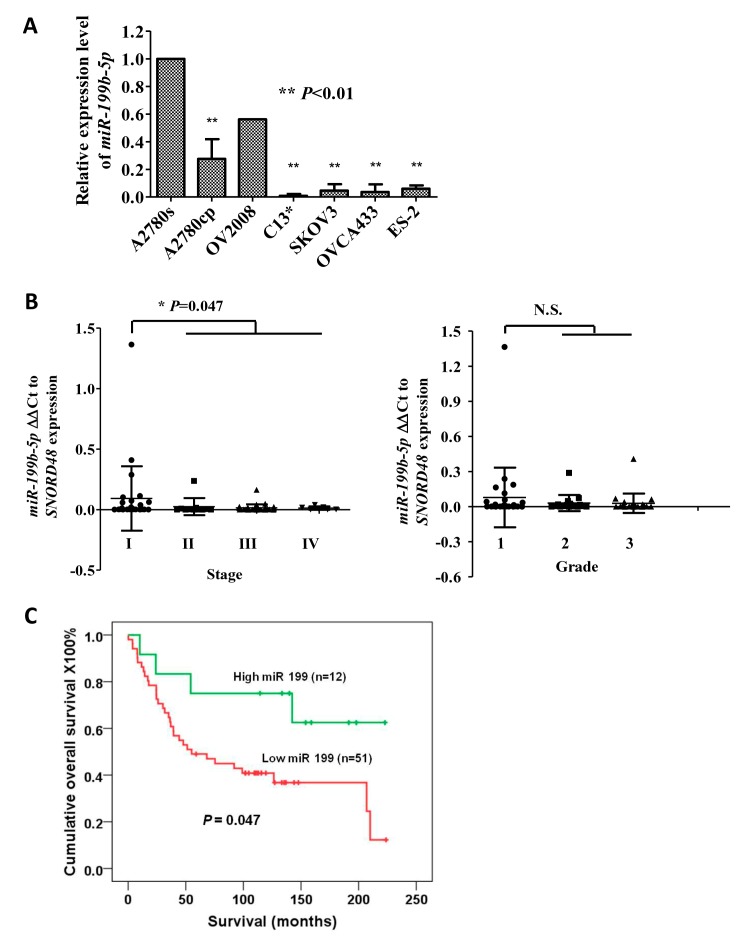
Loss of miR199b-5p is involved in acquired chemoresistance and advanced-stage ovarian cancer (A) miR-199b-5p expression was measured by quantitative RT-PCR in a panel of ovarian cancer cell lines: A2780s, A2780cp, OV2008, C13*, SKOV3, OVCA429, OVCA433, and ES-2. The results are reported as the mean ± SD of three independent experiments performed in triplicate, ** P<0.01. (B) Comparison of miR-199b-5p expression in early-stage (Stage I, N=28) vs. advanced-stage (Stage II, III and IV, N =51) ovarian cancer (P=0.047) and in low-grade vs. high-grade disease as determined by Q-PCR. (C) Comparison of the survival curve of ovarian cancer patients with high (n=12) and low levels (n=51) of miR-199b-5p. The relative expression level of miR-199b-5p (0.024) was used as a cutoff point. * P = 0.047

To investigate the dysregulated miRNAs in ovarian cancer with acquired chemoresistance, microRNA profiling was performed using the miRCURY LNA™ miRNA array on two pairs of ovarian cancer cell lines (cisplatin-sensitive: A2780s and OV2008 vs. cisplatin-resistant: A2780cp and C13*) [15]. Our results revealed that there were 35 upregulated miRNAs (>2-fold) and 45 downregulated miRNAs (<0.5-fold) with statistical significance (data not shown). Among these dysregulated miRNAs, 21 upregulated miRNAs and 10 downregulated miRNAs were commonly found in both pairs of cell lines (Supplementary Fig. S1). In this study, the downregulated miRNAs in chemoresistant ovarian cancer cells were studied. Of 10 downregulated miRNAs, 5 miRNAs (miR-10b, miR-99a, miR-193a-3p, miR-199b-5p and miR-675) were selected for verification of the response to cisplatin-induced cell cytotoxicity via transient transfection of their pre-miRNA-expressing plasmids. Among these 5 candidates, XTT cell proliferation assay demonstrated that miR-199b-5p exhibited a remarkable increase in the cisplatin-mediated cell cytotoxicity of A2780cp and C13* cells (data not shown). As miR-199b-5p could sensitize ovarian cancer cells to cisplatin-induced cytotoxicity, we hypothesized that the loss of the miR-199b-5p is involved in chemoresistance of ovarian cancer. To validate this hypothesis, Q-PCR was performed to evaluate the expression of miR-199b-5p. We confirmed that miR-199b-5p was downregulated in chemoresistant ovarian cancer cells A2780cp and C13* compared with their chemosensitive counterparts A2780s and OV2008 (Fig. 1A). In addition to these cell lines, the expression of miR-199b-5p was also reduced in other chemoresistant ovarian cancer cell lines: SKOV3, OVCA433 and ES-2 [16-18] (Fig. 1A). We observed a trend of progressively diminishing miR-199b-5p expression (measured as 2-ΔCt) from early to advanced stages and from low to high grade ovarian cancer (Fig. 1B). Importantly, the downregulated miR-199b-5p was significantly correlated with poor survival rate (P=0.047) (Fig. 1C). These results indicate that the expression of miR-199b-5p is gradually silenced in ovarian cancer progression and that downregulated miR-199b-5p is significantly associated with chemoresistance and poor survival of ovarian cancer patients.

### Loss of miR-199b-5p is attributed to promoter hypermethylation in ovarian cancer

**Fig 2:**
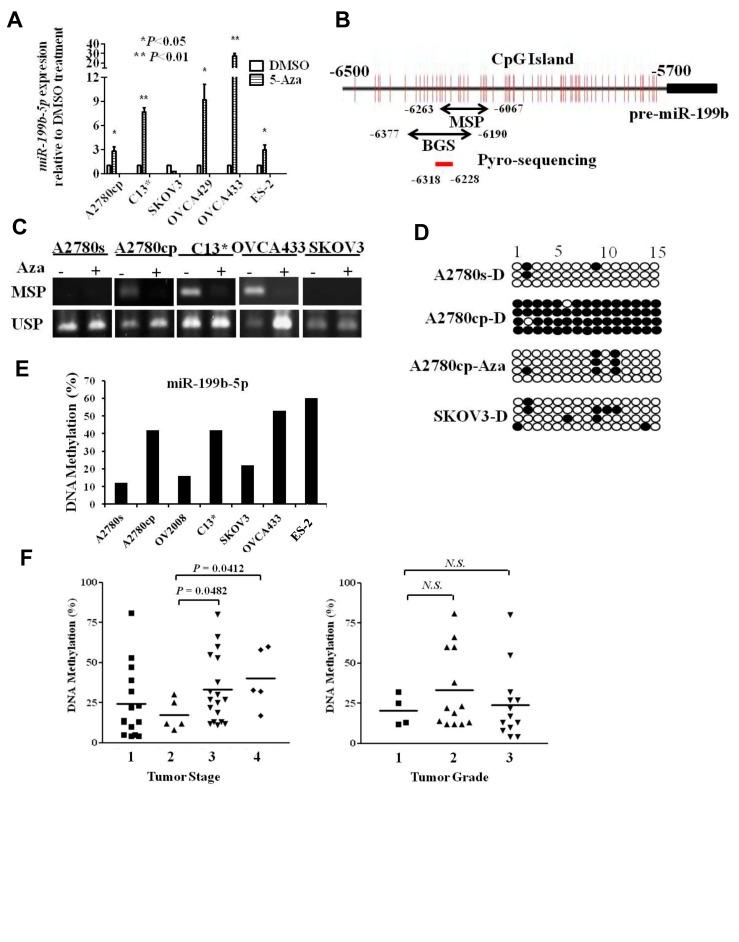
Hypermethylation silences miR-199b-5p expression in ovarian cancer (A) The expression of miR-199b-5p could be restored in cisplatin-resistant ovarian cell lines upon 5-Aza-dC mediated demethylation treatment (*P<0.05, ** P<0.01). (B) Schematic illustration of CpG islands in the promoter region of miR-199b-5p. Vertical lines indicate CpG sites. The arrows indicate the MS-PCR, BGS and pyrosequencing (-6318 to -6228) regions. (C) Promoter methylation status of miR-199b-5p was evaluated by MS-PCR in ovarian cancer cells upon treatment of 5-Aza-dC-mediated demethylation. (D) BGS evaluation of the promoter methylation status of miR-199b-5p in ovarian cancer cells A2780s, A2780cp and SKOV3 with/without 5-Aza-dC mediated demethylation. Each row of circles indicates a single clone. Each circle indicates a CpG site. Filled, methylated CpG site. Unfilled, unmethylated CpG site. (E) Pyrosequencing analysis revealed that methylation density of ovarian cancer cell lines. (F) Pyrosequencing results of ovarian cancer samples indicated that there was a progressive increase in the methylation status with tumor stage (stage 3 vs. stage 2, P=0.0482; stage 4 vs. stage 2, P=0.0412), whereas there was no significance relationship between the methylation status and tumor grade. Each dot represents the methylation percentage of each clinical sample.

Recent evidence suggests that DNA methylation typically silences expression of miRNAs in cancer progression [19, 20]. To investigate whether the downregulation of miR-199b-5p was attributed to promoter hypermethylation in ovarian cancer, 6 ovarian cancer cell lines were treated with the DNA demethylation reagent 5-Aza-2'-deoxycitidine (5-Aza-dc). Q-PCR analysis demonstrated that the expression of miR-199b-5p was significantly restored in 5 out of 6 ovarian cancer cell lines after 5-Aza-dc mediated demethylation (Fig. 2A). Further analysis of the methylation status of the promoter region of miR-199b-5p was performed by bisulfite genomic sequencing (BGS) and methylation-specific PCR (MS-PCR). A CpG island located -6,500 to -5,000 bp upstream of pre-miR-199b was identified (Fig. 2B). MS-PCR and BGS were employed to evaluate the DNA methylation status within the CpG islands of the promoter region of miR-199b-5p in ovarian cancer cell lines. The MS-PCR results revealed that an increase in DNA methylation was frequently observed in the promoter region of miR-199b-5p in chemoresistant ovarian cancer cell lines (A2780cp, C13* and OVCA433), whereas increased DNA methylation status could be reversed with 5-Aza-dc treatment (Fig. 2C). In contrast, less DNA methylation was observed in chemosensitive A2780s cells and in SKOV3 cells in which the expression of miR-199b-5p could not be restored by 5-Aza-dc demethylating activity (Fig. 2C). The DNA methylation status of the miR-199b-5p promoter was further validated by BGS and pyrosequencing analyses, and the results were consistent with the above findings (Figs. 2D and 2E). In clinical samples, pyrosequencing analysis also revealed that DNA methylation was gradually increased in parallel with the tumor stage (Stage 2 vs. Stage 3, P = 0.0482; Stage 2 vs. Stage 4, P = 0.0412) (Fig. 2F). However, due to the small patient cohort, a significant correlation between DNA methylation and tumor grade could not be found (Fig. 2F). Collectively, these data suggest that the downregulation of miR-199b-5p is attributed to increased DNA methylation during repetitive chemotherapeutic challenges and ovarian cancer progression.

### Loss of miR199b-5p is associated with cisplatin-resistant ovarian cancer cells

**Fig 3:**
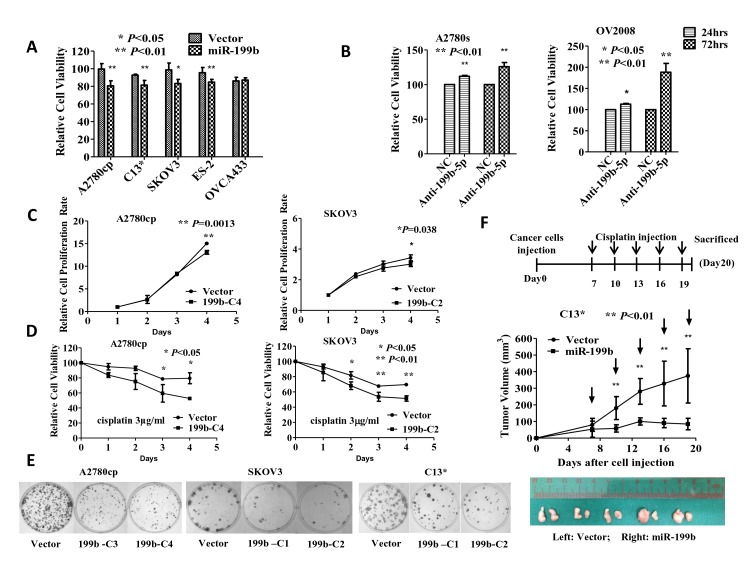
MiR-199b-5p sensitizes chemoresistant ovarian cancer cells to cisplatin-induced cytotoxicity in vitro and in vivo (A) XTT cell proliferation assay revealed that ectopic expression of miR-199b-5p significantly reduce cell viability from 95-99% to 80-85% in A2780cp, C13*, SKOV3 and ES-2 cells upon cisplatin treatment (3 μg/ml, 24 hours). (B) XTT cell proliferation assay revealed that depletion of miR-199b-5p increased cell viability from 11% to 25% in A2780s cells and 12% to 88% in OV2008 upon cisplatin treatment (1 μg/ml) at 24 h and 72 h compared with the negative control. (C & D) XTT cell proliferation assay demonstrated that the stable expression of miR-199b-5p inhibited cell proliferation by 15% in A2780cp cells and 13% in SKOV3 cells but further inhibited the cell viability 19-26% in A2780cp and 13-18% in SKOV3 upon cisplatin treatment (3 μg/ml). (E) The focus formation assay revealed that stable expression of miR-199b-5p caused a 40% reduction in foci formation in C13* and SKOV3 cells and an 80% reduction in A2780cp cells upon cisplatin treatment (3 μg/ml, 14 days). The results are reported as the mean ± SD of three independent experiments performed in triplicate, *, P<0.05, **, P<0.01. (F) miR-199b-5p stable expression (C2) and vector control C13* cells were inoculated subcutaneously into nude mice. The mice were i.p. injected with cisplatin (5 mg/kg) every three days beginning on day 7 when the palpable tumor was formed. The tumor size was calculated and reported as the mean tumor volume ± SE of each group of five mice. The miR-199b-5p stable expression transfectant (C2) resulted in a significantly slower tumor growth rate compared with the vector control. Vertical arrows indicate the time of cisplatin injection. Points, mean of 5 mice. Bars, SD. N=5, ** P<0.01.

To study the functional role of miR-199b-5p in the chemoresistance of ovarian cancer, the pre-miR-199b-5p expression plasmid (pmR-ZsGreen1-miR-199b-5p) was transiently transfected into five chemoresistant ovarian cancer cells (A2780cp, C13*, SKOV3, ES-2 and OVCA433). Upon treatment with cisplatin (3 μg/ml), the XTT cell proliferation assay revealed that enforced expression of miR-199b-5p significantly reduced cell viability by 15-20% in A2780cp, C13*, SKOV3 and ES-2 cells compared with their vector controls (Fig. 3A). In contrast, depletion of miRNA-199b-5p in A2780s and OV2008 by a specific miRNA inhibitor (Ambion Product ID, AM10553) (Supplementary Fig. S2a) dramatically increased cell viability from 25% to 88% in A2780s and OV2008 cells, respectively, compared with their controls upon cisplatin treatment (Fig. 3B).

To study whether miR-199b-5p is functionally relevant in the inhibition of ovarian cancer cell growth, stable expression of miR-199b-5p in A2780cp and SKOV3 cells (Supplementary Fig. S2B) was observed suppressed cell proliferation by 15% in A2780cp and by 13% in SKOV3 cells (Fig. 3C). Furthermore, upon treatment with cisplatin, the miR-199b-5p stably expressing cells in A2780cp (C4) and SKOV3 (C2) exhibited reduced cell viability, approximately 15-20% compared with the vector controls (Fig. 3D). By focus formation assay, the miR-199b-5p stably expressing cells (A2780cp C3 and C4, C13* C1 and C2, SKOV3 C1 and C2) exhibited 40-80% reduced foci formation in both number and size upon cisplatin treatment (Fig. 3E) (Supplementary Fig. S2C). These results indicate that miR-199b-5p is capable of suppressing ovarian cancer cell growth and sensitizing ovarian cancer cells to be cisplatin-induced cytotoxicity.

To confirm the role of miR-199b-5p in enhancing the sensitivity of ovarian cancer cells to cisplatin-mediated cytotoxicity *in vivo*, the miR-199b-5p stably expressing ovarian cancer cell C13* (C2) and vector control were subcutaneously (s.c.) injected into the right and left flanks of mice, respectively. The tumor-bearing mice were then treated with 5 mg/kg cisplatin every three days beginning on Day 7. On Day 19, the size of tumor from the miR-199b-5p expressing cells was profoundly smaller (~70%) than the vector control (Fig. 3F). Taken together, these data suggest that the loss of miR-199b-5p is associated with increased tumor growth and the cisplatin resistance of ovarian cancer cells.

### JAG1 is a direct target of miR199b-5p in ovarian cancer

**Fig 4:**
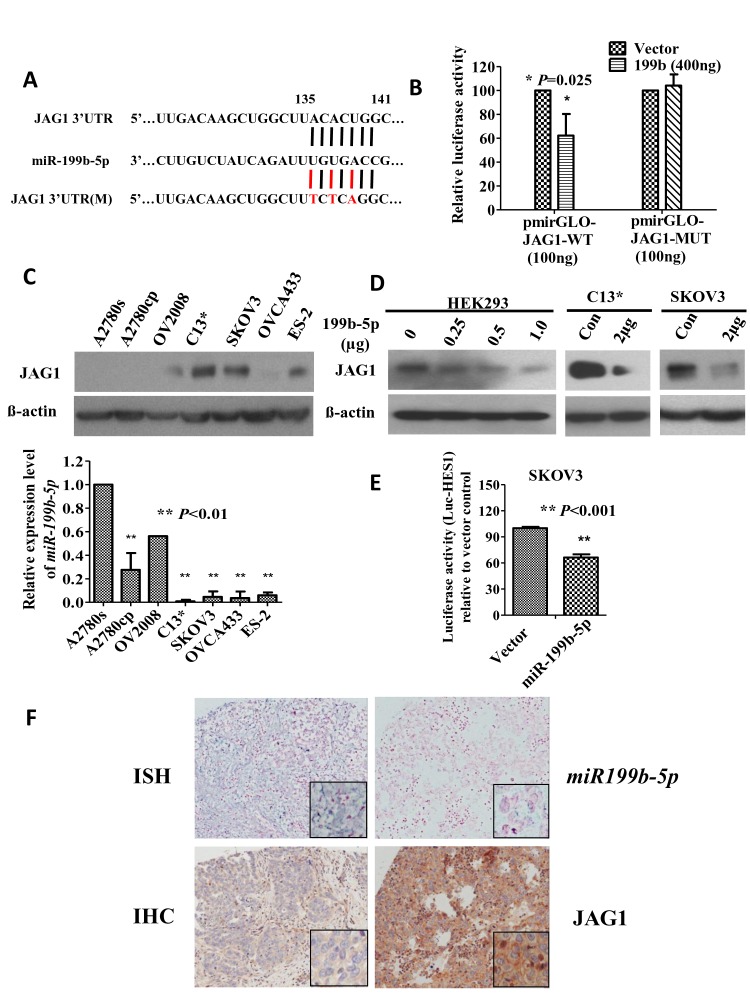
JAG1 is a direct target of mir199b-5p and a key factor mediating Notch1 signaling activity in chemoresistant ovarian cancer (A) A schematic diagram indicating the wild-type and mutated type binding sites of miR-199b-5p in the JAG1 3'UTR. (B) The luciferase reporter assay demonstrated that co-transfection of the JAG1 3'UTR wild-type pmir-GLO-JAG1-WT but not the mutated pmirGLO-JAG1-MUT plasmids (100 ng) with pmR-ZsGreen1-miR-199b-5p (400 ng) resulted in a 38% reduction in the luciferase activity compared with the vector control. *, P=0.025. (C) Both western blot and Q-PCR results indicated a reciprocal expression pattern of JAG1 and miR-199b-5p in a panel of ovarian cancer cell lines. (D) Transient transfection of pmR-ZsGreen1-miR-199b-5p reduced the expression of JAG1 in HEK293 cells in a dose–dependent manner and in chemoresistant C13* and SKOV3 cells. (E) Co-transfection of miR-199b-5p reduced HES1 luciferase activity by 35% compared with the vector control in SKOV3, expressing a relatively higher level of JAG1. (F) Representative pictures showing an inverse relationship between the expression levels of miR-199b-5p and JAG1 examined by ISH and IHC analyses in a commercial ovarian cancer tissue array (OVC1021, N=97, P=0.013). Two-fold and 5-fold changes in expression were used as cutoff level for miR-199b-5p and JAG1, respectively, in the statistical analysis.

To identify the potential targets regulated by miR-199b-5p, bioinformatic databases such as TargetScan and miRanda were searched. Among the list of potential targets, JAG1 was identified as a target of miR-199b-5p. JAG1 contains a highly conserved miR-199b-5p binding site at nucleotides 135—141 in its 3'UTR and is a trans-membrane protein acting as a key ligand of Notch receptors in ovarian cancer [16, 21]. To further prove whether JAG1 acts as a primary target of miR-199b-5p, luciferase reporter plasmids containing the JAG1 3'UTR wild-type binding sites pmirGLO-JAG1-3'UTR-WT (ACACUGG) and the mutated binding sites pmirGLO-JAG1-3'UTR-MUT (TCTCAGG) (Fig. 4A) were constructed and co-transfected with an miR-199b-5p mimic (pmR-ZsGreen1-miR-199b-5p) in HEK293 cells. The result demonstrated that co-transfection of pmirGLO-JAG1-3'UTR-WT and pmR-ZsGreen1-miR-199b-5p could reduce luciferase activity by 38% compared with the vector control. In contrast, co-transfection of pmirGLO-JAG1-3'UTR-MUT and pmR-ZsGreen1-miR-199b-5p exhibited no difference in luciferase activity compared with the vector control (Fig. 4B). Moreover, western blot analysis revealed that the expression of JAG1 was upregulated and was inversely associated with the decreased expression level of miR-199b-5p in ovarian cancer cell lines such as C13*, SKOV3 and ES-2 (Fig. 4C). Enforced expression of miR-199b-5p significantly and dose dependently reduced the expression of JAG1 in HEK293 cells as well as in two chemoresistant ovarian cancer cell lines, C13* and SKOV3 (Fig. 4D). Intriguingly, the enforced expression of miR-199b-5p profoundly reduced HES1 luciferase reporter activity in SKOV3 cells by 35% (Fig. 4E), indicating the JAG1-Notch1 signaling cascade could be suppressed by miR-199b-5p in ovarian cancer cells. These findings suggest that JAG1 is a direct target of miR-199b-5p and that reduced miR-199b-5p and increased JAG1 are involved in ovarian cancer chemoresistance.

To verify whether the upregulation of JAG1 was attributed to the loss of miR-199b-5p in ovarian cancer, both *in situ* hybridization (ISH) and immunohistochemistry (IHC) were performed to examine the expression level of miR-199b-5p and JAG1, respectively, on a commercial human ovarian cancer tissue array (OVC1021, Pantonmics). The results revealed that low expression of miR-199b-5p (≤2-fold) was significantly correlated with high expression of JAG1 (>5-fold) (Fig. 4F, N=97, P=0.013). Intriguingly, clinicopathological analysis revealed that both low expression of miR-199b-5p (≤2-fold) and high expression of JAG1 (>5-fold) were significantly correlated with high-grade tumors (p=0.014 for miR-199b-5p and P<0.001 for JAG1) (Table 1). These results further indicate that the downregulation of miR-199b-5p contributes to the upregulation of JAG1 and is involved in aggressive ovarian cancer, such as high-grade tumors.

**Table 1:** Clinicopathological correlation of the expression of JAG1 in an ovarian cancer tissue array (OVC1021)

Parameters	n(=97)	JAG1 expression		
		≤5-fold	>5-fold	p-value
Grade				
Low (1+2)	50	23 (46%)	27 (54%)	
High (3)	46	4 (9%)	42 (91%)	<0.001*
Stage				
Early (1+2)	73	18 (25%)	55 (75%)	
Late (3)	24	10 (42%)	14 (58%)	0.126
Metastasis				
Yes	24	11 (46%)	13 (54%)	
No	73	17 (23%)	56 (77%)	0.042*

**Table 2:** Clinicopathological correlation of the expression of miR199b-5p in an ovarian cancer tissue array (OVC1021)

Parameters	n(=97)	miR199b-5p expression		
		≤2-fold	>2-fold	p-value
Grade				
Low (1+2)	50	18 (36%)	32 (64%)	
High (3)	46	29 (63%)	17 (37%)	0.014*
Stage				
Early (1+2)	73	38 (52%)	35 (48%)	
Late (3)	24	10 (42%)	14 (58%)	0.482
Metastasis				
Yes	24	10 (42%)	14 (58%)	
No	73	38 (52%)	35 (48%)	0.482
JAG1				
< 5 folds	28	8 (29%)	20 (71%)	
> 5 folds	69	40 (58%)	29 (42%)	0.013*

### JAG1 is required for ovarian cancer cell growth and cisplatin-induced cytotoxicity

**Fig 5:**
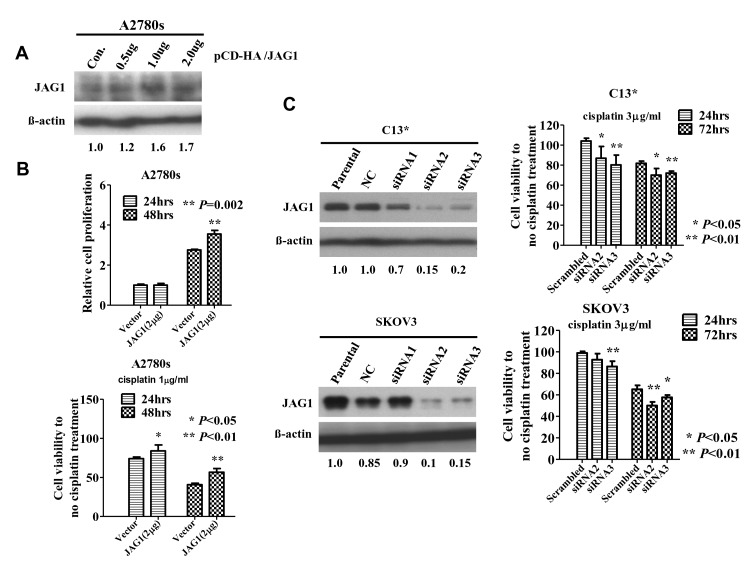
JAG1 enhances ovarian cancer cell growth and cisplatin-resistance (A) Western blot analysis demonstrating the transient transfection of JAG1 (pCD-HA/JAG1) in A2780s cells. (B) XTT cell proliferation assay demonstrated that enforced expression of JAG1 increased the cell proliferation rate by 29% and increased cell viability by up to 40% in A2780s cells upon cisplatin treatment (1 μg/ml). (C) Western blot analysis revealed that two out of three siRNAs (siRNA2 and siRNA3) remarkably reduced JAG1 expression by 80-85% in C13* and 70-75% in SKOV3 cells compared with scrambled controls (NC) (left panel). XTT cell proliferation assay demonstrated that depletion of JAG1 by siRNA reduced cell viability by 15-20% in C13* cells and 10-15% in SKOV3 cells upon cisplatin treatment (3 μg/ml) (right panel). The results are expressed as the mean ± SD of three independent experiments performed in triplicate. *, P<0.05, **, P<0.01.

Aberrant upregulation of JAG1 has been reported to be associated with human cancer development and progression [16, 22, 23]. To study the function of JAG1 in ovarian cancer oncogenesis and chemoresistance, we evaluated the expression of JAG1 on a panel of ovarian cancer cell lines. Western blot analysis demonstrated that JAG1 was upregulated in most ovarian cancer cell lines (Fig. 4C). Intriguingly, higher JAG1 expression was exclusively observed in cisplatin–resistant ovarian cancer cell lines (C13*, SKOV3 and A2780cp) compared with their corresponding cisplatin-sensitive cell lines (OV2008 and A2780s) (Fig. 4C). A long exposure time in western blotting revealed that A2780cp cells exhibited a higher expression level of JAG1 than did A2780s (Supplementary Fig. S3A). Importantly, the higher expression of JAG1 in A2780cp was accompanied by higher levels of Notch1, suggesting that Notch1 signaling activity was higher in A2780cp (Supplementary Fig. S3B). To examine the effect of JAG1 on cisplatin-resistance, one of the cisplatin-sensitive ovarian cancer cell lines, A2780s, was transiently transfected with JAG1 at variable doses (0.5, 1.0, and 2.0 μg) (Fig. 5A). An XTT cell proliferation assay revealed that enforced expression of JAG1 could significantly increase the cell proliferation rate by 29% and enhance the cell viability by 40% upon treatment with cisplatin compared with the vector control (Fig. 5B). Conversely, two chemoresistant cell lines, C13* and SKOV3, expressing a relatively higher level of JAG1 were selected for siRNA-mediated knockdown. An XTT cell proliferation assay indicated that the cell viability was remarkably reduced by 15-20% in C13* cells and by 10-15% in SKOV3 cells upon cisplatin treatment compared with their scrambled controls (Fig. 5C). These data suggest that JAG1 could enhance the cell proliferation and cell viability of ovarian cancer cells to cisplatin-induced cell cytotoxicity.

### JAG1 enhances the chemoresistance of ovarian cancer cells by activating Notch1 signaling activity

**Fig 6:**
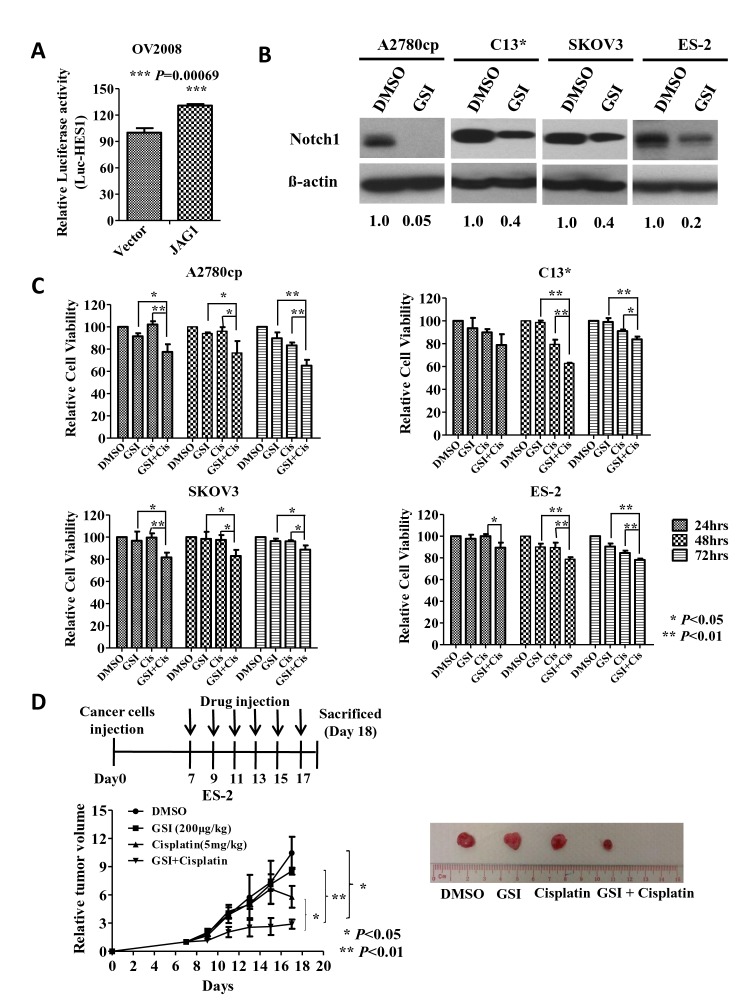
JAG1 enhances the chemoresistance of ovarian cancer cells by activating Notch1 signaling activity (A) HES1 luciferase reporter assay demonstrated that JAG1 was able to increase HES1 luciferase activity by 30% compared with the vector control in OV2008. (B) Western blot analysis revealed that GSI inhibited the expression of the Notch1 active domain by 60-95% in A2780cp, C13*, SKOV3 and ES-2 cells. (C) XTT cell proliferation assay demonstrated that GSI or cisplatin reduced cell viability by 5-10% in A2780cp, C13*, SKOV3 and ES-2 cells after 24 to 72 hrs. Co-treatment with GSI and cisplatin dramatically reduced cell viability by 17-38% in the above cell lines after 24 hrs to 72 hrs when compared with the DMSO control. Dose of DMSO, 10 μM; GSI, 10 μM; cisplatin, 3 μg/ml; GSI, 10 μM, + cisplatin, 3 μg/ml. The results are reported as the mean ± SD of three independent experiments performed in triplicate. *, P<0.05, **, P<0.01. (D) Blocking Notch1 signaling by GSI enhances cisplatin-mediated cell cytotoxicity in a tumor xenograft mouse model. ES-2 cells were injected subcutaneously into nude mice. The tumor-bearing nude mice were separated into 4 test groups on day 7 and were i.p. injected with DMSO, GSI (200 ng/kg), cisplatin (5 mg/kg) or a combination of GSI and cisplatin every two days. The tumor size was monitored and calculated as the mean tumor volume ± SE of each group. The group receiving both GSI and cisplatin exhibited the slowest tumor growth rate. The representative picture shows the tumor size of each group. Vertical arrows indicate the time of drug injection. Points show the mean of 3 mice of each group. Bars, SD. N=3, * P<0.05; ** P<0.01.

JAG1 acts as a key ligand of the Notch1 receptor in cancer development [22, 24, 25]. More importantly, Notch1 signaling has been shown to be involved in chemoresistance in numerous human cancers [26-28]. Hence, we speculated that JAG1-mediated Notch1 signaling activity is involved in ovarian cancer oncogenesis and chemoresistance. To this end, a luciferase reporter assay for the JAG1 expression plasmid (pCD-HA/JAG1) and the HES1 luciferase reporter plasmid (pHES1-luc) [29] was performed in OV2008 cells. HES1 is a well-known downstream target of the Notch1 receptor, and the activation of HES1 represents the activity of Notch1 signaling [30]. The luciferase reporter assay revealed that transient transfection of JAG1 (1 μg) increased HES1 luciferase activity by 30% in OV2008 cells, suggesting that JAG1 is capable of activating Notch1 signaling activity (Fig. 6A).

To further investigate the functional role of Notch1 signaling in the chemoresistance of ovarian cancer, a γ-secretase inhibitor (GSI XXI, compound E, 10 μM) was used to inhibit Notch1 activity in the chemoresistant ovarian cancer cells A2780cp, C13*, SKOV3 and ES-2 (Fig. 6B). Upon treatment with either GSI or cisplatin alone, there was a 5-10% reduction in the cell viability compared with the DMSO control in four ovarian cancer cell lines: A2780cp, C13*, SKOV3 and ES-2 cells (Fig. 6C). However, when the cells were co-treated with GSI and cisplatin together, we observed the synergistic enhancement of cisplatin-induced cytotoxicity, with a 17-38% reduction in the cell viability of all four ovarian cancer cell lines (Fig. 6C). To further explore whether blocking Notch1 signaling could enhance cisplatin-mediated cell cytotoxicity *in vivo*, 5-week BALB/cAnN nude mice were subcutaneously injected with the chemoresistant ovarian cancer cell line ES-2. When the tumors were palpable on Day 7, the mice were randomly divided into 4 groups. Each group was administered one treatment by intraperitoneal injection every two days: DMSO, GSI (200 μg/kg), cisplatin (5 mg/kg) or GSI (200 μg/kg) combined with cisplatin (5 mg/kg). When the mice were treated with GSI or cisplatin alone, the tumor growth was inhibited by 20% and 45%, respectively, compared with DMSO group (Fig. 6D). However, co-treatment with GSI and cisplatin synergistically enhanced cisplatin-induced cytotoxicity, resulting in a 73% reduction in tumor growth on day 18 (Fig. 6D). These results indicate that the JAG1/Notch1 signaling pathway is indispensable for cisplatin resistance in ovarian cancer cells.

## DISCUSSION

The acquisition of resistance during tumor progression and repeated challenges of chemotherapy is the major obstacle of clinical management of ovarian cancer [15, 31-33]. However, the underlying mechanisms leading to chemoresistance in ovarian cancer remain poorly understood. In this study, we identified that the loss of miR-199b-5p, mediated by increased DNA methylation, is associated with acquired cisplatin resistance via aberrant activation of JAG1-Notch1 signaling in ovarian cancer. Blocking the JAG1-Notch1 signaling axis by either re-expression of miR-199b-5p, siRNA-mediated JAG1 knockdown or treatment with the Notch-specific inhibitor γ-secretase (GSI) dramatically abrogated tumor growth and enhance cisplatin-induced cytotoxicity *in vitro* and *in vivo* in ovarian cancer cells. Our study provides a novel regulatory circuit in which the epigenetic machinery silences the expression of miRNAs to dysregulate the activity of the key signaling pathways involved in developing ovarian cancer chemoresistance.

Mounting evidence has suggested that the dysregulation of miRNAs causes aberrant upregulation of oncogenes or the downregulation of tumor suppressor genes which in turn leads to cancer initiation and progression [34-36]. In this study, miR-199b-5p was identified as a downregulated miRNA from two pairs of cisplatin sensitive vs. resistant ovarian cancer cell lines (A2780s vs. A2780cp and OV2008 vs. C13*) [15, 37, 38]. The cisplatin-resistant cell lines A2780cp and C13* were obtained from repetitive challenges of cisplatin treatment to A2780s and OV2008 cells. This repeated challenge of cisplatin treatment mimics the clinical situation in which the patient is repeatedly treated by cisplatin before they are in recurrence [39]. Interestingly, the downregulation of miR-199b-5p was progressively enhanced from early to advanced stages and low- to high-grade ovarian cancer tissue samples, indicating that the loss of miR-199b-5p is involved in ovarian cancer progression. Accumulating evidence has documented that aberrant genetic and epigenetic alterations always occur during cancer progression, including in the progression of ovarian cancer [40-42]. These alterations result in abnormal activation of functional signaling, which in turn leads to so-called “acquired chemoresistance”. Hence, acquired chemoresistance is proposed to be a dominant player in ovarian cancer [7, 43-45]. In this study, forced expression of miR-199b-5p could not only suppress ovarian cancer cell growth but also sensitize resistant ovarian cancer to cisplatin treatment *in vitro* and *in vivo*. These results suggest that miR-199b-5p plays a significant role in tumor suppression by inhibiting cell growth and enhancing the chemosensitivity of epithelial ovarian cancer.

MiR-199b-5p was progressively downregulated from early to advanced stage and low- to high-grade ovarian cancer, suggesting that the loss of miR-199b-5p is the gradual result of epigenetic silencing mechanisms such as hypermethylation. Indeed, DNA hypermethylation has been frequently reported to silence tumor suppressor miRNAs in various human cancers, including ovarian cancer [46, 47]. For instance, downregulation of the miR-34 family is induced by promoter hypermethylation and leads to the pathological progression of epithelial ovarian cancer [48]. Our results demonstrate that the expression of miR-199b-5p could be restored in 5 out of 6 ovarian cancer cell lines using 5-Aza-dC-mediated DNA demethylation. MS-PCR, BGS and pyrosequencing analyses further confirmed that an increase in the DNA methylation of the promoter of miR-199b-5p in ovarian cancer cell lines. More importantly, although there is a limited number of ovarian cancer samples for pyrosequencing analysis, our data suggests that there is a progressive increase in DNA methylation that is inversely correlated with the expression of miR-199b-5p during the progression of ovarian cancer. Therefore, these findings support our hypothesis that the loss of miR-199b-5p is attributed to increased DNA methylation during tumor progression and repetitive treatment of chemotherapy in ovarian cancer.

Dysregulation of the JAG1-Notch1 signaling cascade has been shown to activate a wide range of oncogenes in the HES, HEY and MYC families, thereby leading to ovarian cancer progression and chemoresistance [21, 49, 50]. Platinum-based chemotherapy is the standard first-line regimen for ovarian cancer patients with advanced-stage disease. Cisplatin is the most common platinum-based compound capable of causing inter- and intra-strand DNA crosslinks that induces cell apoptosis with DNA damage [51, 52]. Previous studies have proposed that the aberrant activation of the JAG1-Notch1 signaling pathway may protect cancer cells from cisplatin-induced cell apoptosis and that the signaling activity is inversely correlated with the cisplatin sensitivity of human cancers [53-57]. Moreover, Notch signaling is associated with tumor growth and angiogenesis [58, 59]. A recent study has suggested that the modulation of tumor angiogenesis by blocking VEGF signaling could reduce the chemoresistance of ovarian cancer [60], suggesting that JAG1/NOTCH1/neovascularization confers ovarian cancer chemoresistance. Indeed, our work provides compelling evidence that overexpressed JAG1 could enhance ovarian cancer cell proliferation, whereas depletion of JAG1 by siRNAs or suppression of Notch1 signaling by γ-secretase inhibitor (GSI) could sensitize chemoresistant ovarian cancer cells to cisplatin-induced cytotoxicity by *in vitro* and *in vivo* models, suggesting that the JAG1-Notch1 signaling axis plays a critical role in the development of ovarian cancer chemoresistance.

Recent studies have reported that the expression of JAG1 is modulated by miRNAs. For instance, JAG1 could be repressed by miR-34b in estrogen-dependent breast cancer cells [61] and by miR-524-5p in glioma [62]. In this study, miR-199b-5p was identified as another novel miRNA regulating the expression of JAG1 in ovarian cancer. We demonstrated that the forced expression of miR-199b-5p could suppress ovarian cancer cell growth and sensitize the cells to cisplatin-induced cytotoxicity. On the other hand, as a direct target of miR-199b-5p in ovarian cancer cells, JAG1 depletion by siRNAs also resulted in cell growth retardation and sensitization to cisplatin-induced cytotoxicity. In contrast, activating Notch1 signaling by JAG1 or repressing miR-199b-5p by anti-miR-199b-5p could induce the activity of JAG1-Notch1 signaling in ovarian cancer cells. Therefore, this study suggests that the loss of miR-199b-5p increased the activation of JAG1-Notch1 signaling, which in turn promoted ovarian cancer progression and acquired chemoresistance. Thus far, this is the first report, to our knowledge, that the miR-199b-5p-JAG1-Notch1 signaling pathway is a novel regulatory circuit for acquired chemoresistance in ovarian cancer.

In conclusion, the findings in this study enhance our understanding of acquired chemoresistance in ovarian cancer during tumor progression or repetitive platinum-based chemotherapy. The epigenetic silencing of miR-199b-5p may at least partly activate JAG1-Notch1 signaling activity and promote acquired chemoresistance in ovarian cancer. Therefore, targeting the miR-199b-5p-JAG1-Notch1 regulatory circuit might be a therapeutic approach in cisplatin-resistant ovarian cancer.

## MATERIALS AND METHODS

### Cell lines and clinical samples

Ovarian cancer cell lines (A2780s, A2780cp, OV2008, C13*) (kindly provided by Prof. Benjamin K. Tsang, Department of Obstetrics and Gynecology, University of Ottawa), and SKOV3, OVCA433 and ES-2 (purchased from American Type Culture Collection, ATCC) used in this study were grown at 37°C with DMEM medium supplemented with 10% fetal bovine serum (FBS). Fresh snap-frozen surgical specimens of tumor tissues from 79 EOC patients completely resected at the Queen Mary Hospital (Hong Kong) were consecutively collected. All the clinical specimens used in the present study were approved by the local institutional ethics committee (IRB: UW 11-298).

### Plasmids and Drugs

The pmR-ZsGreen1-miR-199b-5p expressing construct (Forward- TCTCAGCCCAAGCTTCCGCTC, Reverse- AATGTGAGTGGATCCTTGCAC, 657bp) was amplified and ligated into pmR-ZsGreen1 plasmid (Clontech, Mountain View, CA). The miRNA inhibitor targeting miR-199b-5p was purchased from Applied Biosystems (Product ID: AM10553, Applied Biosystems, Foster city, CA). The small interfere RNAs (Duplex Oligonucleotide) targeting JAG1 were purchased from IDT (Integrated DNA Technologies, Coralville, USA) (Supplementary Table 1). The JAG1 expressing plasmid (pCD-HA/JAG1) used for JAG1 expression was provided by Prof. Aly Karsan (British Columbia Cancer Research Centre, Canada), and pHES1-luc plasmid used for luciferase reporter assay was provided by Prof. Ryoichiro Kageyama (Kyoto University, Japan). Cisplatin (CDDP) was obtained from Calbiochem (Darmstadt, Germany) and was stocked at a concentration of 5 mg/ml at −20°C. 5-Aza-2'-deoxycytidine (5-Aza-dC, Sigma Chemical Co., St Louis, MD, USA) at a concentration of 20 mM was stored at −20°C and freshly dissolved in culture medium before use. γ–secretase inhibitor (GSI XXI, Compound E, Merck, Darmstadt, Germany) was stocked at a concentration of 1 mM at −20°C and freshly diluted to 10 μM in culture medium before use.

### RNA isolation and quantitative real-time RT-PCR

Total RNA from cell lines and primary cancer tissue samples was prepared by TRIzol reagent (Invitrogen Life Technology). First-strand complementary DNA was synthesized and quantitative reverse transcription–polymerase chain reaction was done using the miRCURY LNA™ Universal RT miRNA PCR Kit (Product No. 203450, Exiqon, Denmark) in an ABI 7500 system (Applied Biosystems). The miR-199b-5p probes were obtained from Exiqon (Product No. 204152). Each sample was performed in triplicate and normalized with human SNORD48 (Product No. 203903, Exiqon, Denmark). Bisulfite conversion, Methylation specific PCR (MS-PCR), Bisulfite Genomic Sequencing (BGS) and pyrosequencing analysis

Genomic DNA was extracted using the DNeasy Blood & Tissue Kit (QIAGEN, Valencia, CA) in ovarian cancer cell lines and cancer samples. The genomic DNA was then applied for bisulfite conversion using the EZ DNA Methylation-Gold Kit (Zymo Research, Orange, California, USA) according to the manufacturer's protocol. The MS-PCR and BGS sequence was amplified by MyTaq™ HS Mix Reaction Buffer (Bioline, London, UK). The BGS amplified PCR products were cloned into the Topo TA vector (Bioline). Colonies were randomly picked for mini preparation and sequencing. The primers and PCR conditions for MSP and BGS are listed in (Supplementary Table 2.) Pyrosequencing assays were designed using PSQ Assay Design Software (version 1.0.6, Biotage). The primers for amplifying the pyrosequencing region of miR-199b-5p were forward 5'- GGGAAGAGTTATGTAAGTGTTGGAAAGA-3' and reverse 5'-AAAACTTCCCCTAACCCTTTC -3'. This assay amplifies a region of 357bp in advanced of miR-199b-5p (position -6,389 – -6,033), which includes ten CpG sites to be analyzed. These ten CpG sites were included in the BGS region.

### Cell viability assays

Cell viability was evaluated by XTT assay and Focus formation assay. Approximately 2,000—3,000 cells were seeded into each well of a 96-well plate. The medium was refreshed 24 hours after cell seeding containing appropriate concentration of cisplatin or γ–secretase inhibitor. XTT was measured by Cell Proliferation Kit II (Roche Biosciences, Indianapolis, IN, USA) according to the manufacturer's instructions. For focus formation assay, 5000 cells with stable expression of miRNA or control vector were seeded into 6-well plates. The medium was replaced with fresh medium containing appropriate concentration of cisplatin 24 hours after cell seeding and grew for about 14 days during which fresh medium containing cisplatin was refreshed for every 4 days. Cells were stained with 1% crystal violet (Sigma-Alrich, St. Louis, MO, USA). Numbers of foci were counted after the cells dried. Experiments were performed in triplicates and results were expressed as the mean ± SD.

### Luciferase reporter assay

Luciferase constructs were made by ligating oligonucleotides containing the wild-type (WT) or mutant sites (MUT) of the 3'UTR of JAG1 gene (position 135—141) into the multi-cloning site of the pmirGLO plasmid (Promega, Madison, WI) (Supplementary Table 3). HEK293 cells were seeded into a 24-well plate and co-transfected with 100 ng pmirGLO-JAG1 plasmid, pmR-199b plasmid and pmR-ZsGreen1 empty vector for balance (400 ng in total), using Lipofectamine™ 2000 (Invitrogen). Luciferase activity was determined by the Dual-Luciferase Assay Kit (Promega) 48 h after transfection using a Fluorescence Spectrophotometer F-4500 (Promega).

### Western Blot

Cells were harvested and lysed by RIPA Buffer (Sigma Chemical Co., St Louis, MD, USA). Samples containing equal amounts of protein were separated by SDS-PAGE and electroblotted onto Immobilon-P Transfer Membrane (Millipore Corporation, Bedford, MA). The membrane was blocked with 5% no-fat milk, followed by incubation with antibodies specific for anti-JAG1 (1:500, 28H8, Cell Signaling Technology, Beverly, MA), anti-Notch1 (1:1000, C-20,sc-6014, Santa Cruz Biotechnology, Santa Cruz, CA) and anti-β-actin (1:5000, AC-74, Sigma Chemical Co), respectively. Blots were then incubated with goat anti-rabbit or anti-mouse secondary antibody conjugated to horseradish peroxidase (Amersham Pharmacia, Cleveland, OH) accordingly. The signals were captured by FUJI Medical X-Ray Film (Fuji) and developed by the FUJI system.

### Immunohistochemistry (IHC) and *in situ* hybridization (ISH)

The commercial tissue array slide (OVC1021, Pantomics) was deparaffinized in xylene and rehydrated in alcohol, then immersed in sodium citrate buffer (pH6) and boiled in boiled water for 20 minutes. 0.3% hydrogen peroxidase (H_2_O_2_) was used to inhibit endogenous peroxidase. 10% normal rabbit serum was applied for blocking non-specific bindings for 45 minutes. Following this, slides were incubated with primary antibodies anti-JAG1 monoclonal antibody (1:100) at predetermined dilutions at 4°C overnight. 0.1% Tween 20 (TBS-T) was used to wash the slides for three times. Staining was carried out following standard streptavidin-biotin-peroidase complex method. Mayer's hematoxylin was used for counterstained. Two independent investigators were invited to review counterstained slides. Scores were then given to the stained slides according to the intensity and the percentage of the stained tissues.

*in situ* hybridization (ISH) was performed to evaluate the miR-199b-5p expression in a commercial tissue array (OVC1021, Pantomics) using the miRCURY LNA™ microRNA ISH Optimization Kit 5 (FFPE) (Exiqon, Vedbaek, Denmark). In brief, the tissue array slide was deparaffinized and incubated with 20 μg/ml Proteinase-K for 40 minutes at 37°C. Following this, the slide was dehydrated and hybridized with specific miR-199b-5p probe (1:500) at 50°C overnight. After hybridization, the slide was incubated with anti-DIG reagent (sheep anti-DIG-AP at 1:400) for 60 minutes at room temperature and applied to freshly prepared AP substrate and incubated for 2 hours at 30°C in a dark, humidifying chamber. Finally, it was stained for nuclear counter and mounted with mounting medium (Eukitt®). The precipitate was allowed to settle overnight and the results were analyzed by light microscopy on the next day.

### *In vivo* tumorigenicity

Ccancer cells with miR-199b-5p stably expression or vector control were injected subcutaneously into the right and left dorsal flank (2×10^6^ per side), respectively, of BALB/c nude mice (5 mice per group). When the tumors were palpable, cisplatin (5mg/kg, Pharmachemie BV, Netherlands, Platosin, 1mg/ml) was injected into the mice in peritoneal every three days. Tumor size was observed by measuring the tumor volume calculating by the formula V=6/π*[(L+W)/2]^3^. Tumors were then excised after sacrifice. All animal experiments were approved by the Committee on the Use of Live Animals in Teaching and Research of The University of Hong Kong (CULATR 2053-09),

### Statistical analysis

Statistical analysis was carried out using SPSS 14.0 (SPSS). Student's t-test and the Mann–Whitney test were used. The results were expressed as mean ± SD. χ2 test or Fisher's exact test was used to analyze the association of miR-199b-5p and JAG1 gene expression and clinicopathological parameters. Differences were considered significant when p ≤ 0.05.

## SUPPLEMENTARY FIGURES AND TABLES




