# Supplementary material for: Epigenetic silencing of microRNA-199b-5p is associated with acquired chemoresistance via activation of JAG1-Notch1 signaling in ovarian cancer
Source: Oncotarget. 2013 Dec 4;5(4):944–58. doi: 10.18632/oncotarget.1458 (PMC4011596; doi:10.18632/oncotarget.1458)
Supplement: Supplementary file 1 [file oncotarget-05-944-s001.pdf]

Epigenetic silencing of microRNA-199b-5p is associated with acquired chemoresistance via activation of JAG1-Notch1 signaling in ovarian cancer - Liu et al

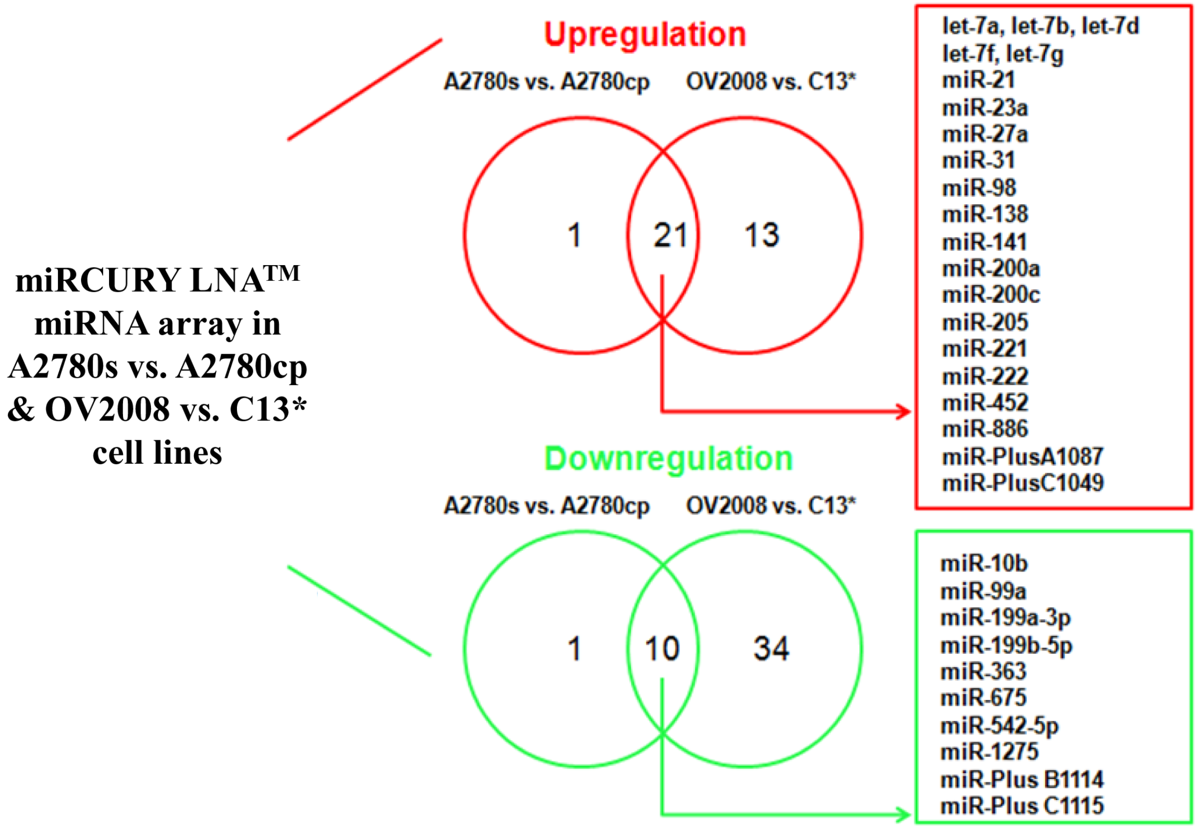

Supplementary Figure S1: Dysregulation of microRNAs obtained from microRNA expression profiling in chemosensitive vs. chemoresistant cell lines (A2780s vs. A2780cp and OV2008 vs. C13*). MiRNAs in the red box represent the 21 miRNAs commonly upregulated in both of the cell lines examined. MiRNAs in the green box indicate the 10 miRNAs commonly downregulated in both of the cell lines examined.

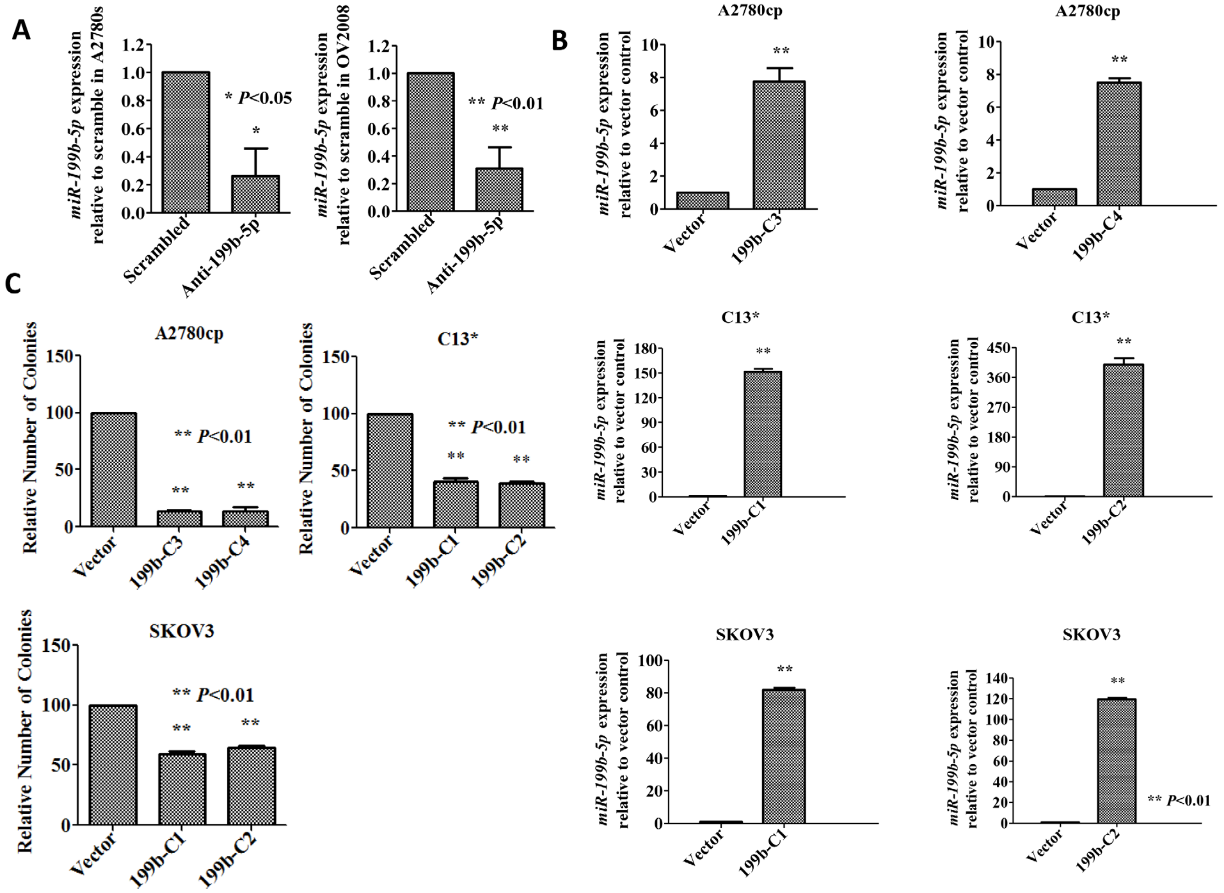

Supplementary Figure S2. (A) Quantitative RT-PCR demonstrated the expression level of miR-199b-5p in A2780s and OV2008 cells treated by anti-miR-199b-5p. (B) Quantitative RT-PCR demonstrated the expression of miR-199b-5p in A2780cp, C13* and SKOV3 after transfection with miR-199b-5p (pmR-ZsGreen1-miR-199b-5p). The empty vector (pmR-ZsGreen1) was used as negative control. (C) Focus formation assay revealed that stable expression of miR-199b-5p led to a 40% reduction in focus formation in C13* and SKOV3 cells and an 80% reduction in A2780cp cells upon treatment with cisplatin (3 µg/ml, 10-14 days). The results are shown as the mean ± SD of three independent experiments performed in triplicate. *, P<0.05, **, P<0.01.

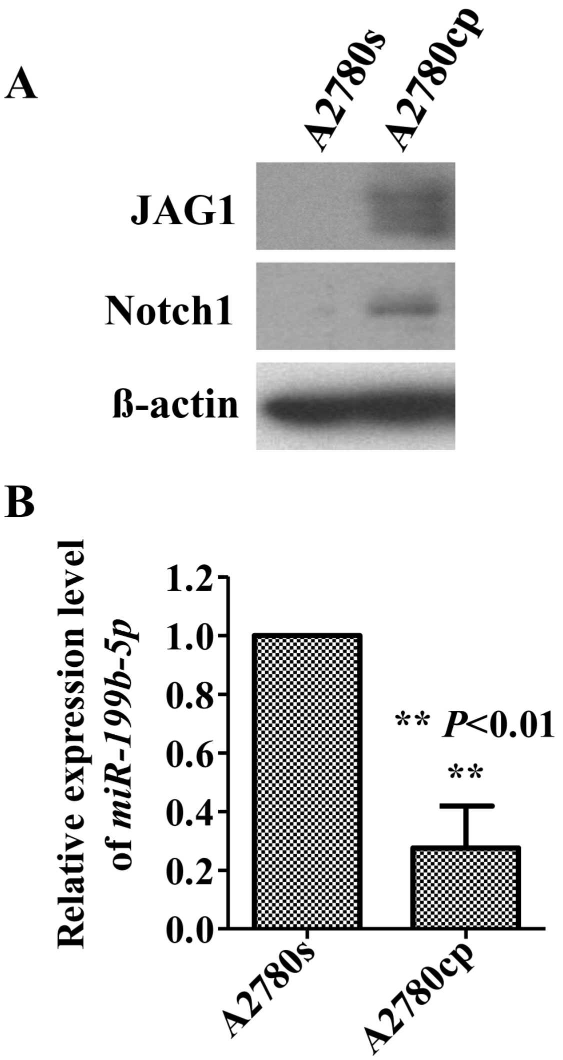

Supplementary Figure S3. An inverse relationship between miR-199b-5p and JAG1 expression is observed in A2780cp cells. (A) Long exposure revealed higher expression of JAG1 in A2780cp cells compared with A2780s in western blotting. Higher levels of Notch1 were accompanied by higher expression of JAG1 in A2780cp cells. (B) miR-199b-5p expression in A2780cp cells compared with the cisplatin-sensitive counterpart A2780s was determined by quantitative RT-PCR (**P<0.01). The results are reported as the mean ± SD of three independent experiments performed in triplicate.
