# Supplementary material for: Epigenetic silencing of microRNA-199b-5p is associated with acquired chemoresistance via activation of JAG1-Notch1 signaling in ovarian cancer
Source: Oncotarget. 2013 Dec 4;5(4):944–58. doi: 10.18632/oncotarget.1458 (PMC4011596; doi:10.18632/oncotarget.1458)
Supplement: Supplementary file 2 [file oncotarget-05-944-s002.pdf]

Epigenetic silencing of microRNA-199b-5p is associated with acquired chemoresistance via activation of JAG1-Notch1 signaling in ovarian cancer - Liu et al

Supplementary Table 1. Sequences of siRNAs (Duplex oligonucleotides) for JAG1

	Sequence
Duplex Sequence 1	5'-GGAACAACCUGUAACAUAGCCCGAA-3'
	3'-UUCCUUGUUGGACAUUGUAUCGGGCUU-5'
Duplex Sequence 2	5'GGAAAUCAAAGUGCUAUUACGAAGT-3'
	3'-CUCCUUUAGUUUCACGAUAAUGCUUCA-5'
Duplex Sequence 3	5'-CCACAGCAACGAUCACAAAUGACTT-3'
	3'-UUGGUGUCGUUGCUAGUGUUUACUGAA -5'

Supplementary Table 2. Primers for MS-PCR, US-PCR and BGS

MS-PCR	Sequence
Forward	ATACGTGGCGTGGTTTCGGTAT
Reverse	AAATACCCTCGCGCCCCAACCG
US-PCP	Sequence
Forward	ATATGTGGTGTGGTTTTGGTAT
Reverse	AAATACCCTCACACCCCAACCA
BGS	Sequence
Forward	GTAAGTGTTGGAAAGAAGGG
Reversed	AAACCCTACTCCTAAACCTC

Supplementary Table 3. Nucleotides for luciferase reporter construct of pmirGLO-JAG1-3'UTR-WT and pmirGLO-JAG1-3'UTR-MUT

**Wild Type**	**Sequence**
**Sense (63bp)**	**CTGTGTTAATTTAAGTTTTGACAAGCTGGCTTACACTGGCAATGGTAGTTTCTGTG GTTGGCT**
**Antisense (71bp)**	**CTAGAGCCAACCACAGAAACTACCATTGCCAGTGTAAGCCAGCTTGTCAAAACTTA AATTAACACAGAGCT**
**Mutant Type**	**Sequence**
**Sense (63bp)**	**CTGTGTTAATTTAAGTTTTGACAAGCTGGCTTTCTCAGGCAATGGTAGTTTCTGTG GTTGGCT**
**Antisense (71bp)**	**CTAGAGCCAACCACAGAAACTACCATTGCCTGAGAAAGCCAGCTTGTCAAAACTTA AATTAACACAGAGCT**

Nucleotides in red color and underlined indicate the binding sites (wild type and mutated type) between miR-199b-5p and 3'UTR of JAG1.
